# Passing tests and using one's attitude to help patients overcome their pathogenic feelings of guilt and shame

**DOI:** 10.1002/jclp.23738

**Published:** 2024-08-27

**Authors:** Francesco Gazzillo, David Kealy, Eleonora Fiorenza, Marta Rodini

**Affiliations:** ^1^ Department of Dynamic and Clinical Psychology, and Health Studies Sapienza University of Rome Roma Italy; ^2^ Department of Psychiatry Divison of Adult Psychiatry and Mental Health Services University of British Columbia Vancouver British Columbia Canada; ^3^ Control‐Mastery Theory – Italian Group Roma Italy

**Keywords:** attitude, Control‐Mastery Theory, guilt, pathogenic beliefs, shame

## Abstract

Guilt and shame are emotions that, albeit subjectively negative, help humans adapt to their social environment. However, in some cases, there are pathogenic beliefs, shaped over the lifespan that sustain them and make them a source of psychopathological suffering. In this paper we will first briefly show how Control‐Mastery Theory (CMT) considers several types of pathogenic beliefs shaped by traumatic experiences that underly chronic feelings of guilt and shame. We then focus on a clinical case of Livia, a 28 year‐old woman with relational and academic problems suffering mainly from two such types of pathogenic beliefs: burdening guilt and disloyalty guilt. We describe how a) Livia was driven by adverse and traumatic experiences to form some of these pathogenic beliefs, b) how she tested the therapist in order to discover whether he would disconfirm these beliefs, and c) how the therapist was able to successfully pass these tests and provide her with new and healthier interpersonal experiences. The case of Livia will highlight therapists' ability to accurately formulate patients' goals, pathogenic beliefs—including types of guilt‐ and shame‐related beliefs—and traumas. Moreover, the case will illustrate how therapists can pass patients' tests and adopt the right attitude to help patients disprove their pathogenic beliefs and overcome problematic experiences of guilt and shame.

## 1 INTRODUCTION

Aspects of guilt and shame have long been recognized to underlie many of the problems that patients seek to address in psychotherapy (Dearing and Tangney, 2011). Indeed, maladaptive variants of these self‐conscious emotions have been linked with psychopathology such as affective disorders (Kim et al., 2011) and suicidality (Sheehy et al., 2019), and as such are critical targets for intervention in psychotherapy.

Recent developments in both psychodynamic thinking about guilt and shame and in developmental and evolutionary psychology (for a review, see Gazzillo et al., 2020) have stressed how guilt and shame are basically adaptive prosocial feelings with roots in universal moral sensitivities that are shaped by actual experiences, teachings, and cultural values. These contemporary views suggest that therapists' attention should be focused on patients' actual adverse and traumatic experiences, particularly from their developmental period (Weiss, 1995), rather than on unconscious fantasies, to understand and treat the beliefs supporting irrational guilt and shame.

Empirical literature clearly shows a close link between pathological shame and a range of mental health problems such as anxiety (Cândea and Szentagotai‐Tătar, 2018), depression (Kim et al., 2011), eating disorders, addictions, borderline and narcissistic personality disorder (e.g., Buchman‐Wildbaum et al., 2021), suicidal ideation and self‐harm (Sheehy et al., 2019). Research also implicates pathological shame in psychological syndromes that follow from traumatic experiences, including post‐traumatic stress disorder (PTSD) and complex PTSD (e.g., DeCou et al., 2023). Moreover, pathological shame is associated with pathological worry and with the tendency to ruminate about one's actions, behaviors, and reactions at the time of the trauma (Gazzillo et al., 2021). From an evolutionary biopsychosocial perspective, shame is associated with feelings of threat to oneself and social status. This threat is perceived by the individual as a result of criticism and rejection, which would endanger one's social attractiveness. From this point of view, shame is seen as an attempt to save oneself from rejection by others, and thus restore positive social relations and a sense of well‐being (Gilbert, 2010). According to the Compassion Focused model (CFT; Gilbert, 2010; Matos et al., 2020) there are two types of shame: *external shame*, that has to do with the belief that one is receiving negative judgments from others due to personal deficits or flaws; *internal shame*, on the contrary, derives from an identification with the negative view that one thinks the other has of the self, is often related to self‐criticism (Gilbert, 2010) and is usually consequent to intense and constant feelings of shame and rejection experienced during childhood and adolescence (Matos et al., 2020).

Research data investigating the relationship between guilt and psychopathology are less certain. Empirical findings (Shi et al., 2021) point out that people who experience chronic guilt are more prone to develop internalizing issues such as anxiety, depression, and PTSD symptoms, but also externalizing symptoms such as anger and hostility. In addition, chronic guilt is related to self‐punishing behaviors in response to events that elicit guilt, and often lead to rumination and self‐sabotaging behaviors. Indeed, maladaptive and/or generalized forms of guilt are more closely linked with psychopathology than episodic or scenario‐based guilt, that may be appropriate to certain contexts (Cândea and Szentagotai‐Tătar, 2018; Kim et al., 2011).

In this paper, we will first focus on how Control‐Mastey Theory (CMT; Gazzillo, 2021, 2023; Silberschatz, 2005; Weiss, 1995) conceptualizes irrational guilt and shame and how they should be addressed in psychotherapy; then we will illustrate these ideas describing the different phases of the therapy of a patient treated by a CMT therapist for guilt related problems.

## 2 THE BASIC CONCEPTS OF CONTROL‐MASTERY THEORY

CMT is an integrative, relational, cognitive‐dynamic theory of mental functioning, psychopathology and psychotherapy process developed by Joseph Weiss and Harold Sampson and empirically validated in the last 50 years by the San Francisco Psychotherapy Research Group and the Control‐Mastery Theory Italian Group.

CMT starts from the assumption that the overarching goal of both conscious and unconscious mental functioning is *adaptation* to reality, i.e. the ability to modify one's own functioning and environment to pursue evolutionary based, adaptive goals. Anticipating recent developments in social cognition and evolutionary psychology, CMT stresses how the human mind is *able to perform unconsciously many of the complex cognitive activities* necessary for adaptation that are usually attributed to consciousness. These functions include setting goals, developing plans to pursue goals, assessing the consequences of their plans, developing and testing beliefs, and making decisions (for a review, see Leonardi et al., 2022). Thus, according to CMT, human beings are intrinsically motivated to *solve their problems and master* ^1^ *adverse experiences (i.e., traumas)* that threaten or interfere with adaptational development. Moreover, CMT hypothesizes that the basic principle that we follow in regulating our mental functioning is a *safety* principle (Fiorenza et al., 2023). In other words, we need to feel safe and we decide (consciously and unconsciously) to do something only when and if we feel safe to do it, or because we think that we could feel safer if we will do it. In using safety as the basis for regulation, we consider both our personal sense of safety and how safe our important others would be if we decided to do something, reflecting the basic *prosociality* of human mental functioning.

To adapt to reality and to feel safe in their environment, human beings from the beginning of life need to establish and maintain both predictable and stable close relationships with their important others (first of all their caregivers and siblings), and a set of beliefs about themselves, their important others and the reality they live in. These beliefs help us foresee the consequences of our actions and anticipate what is needed to reach our goals. Such beliefs can be conscious, explicit, and declarative, or unconscious, implicit, and procedural, “bodily” and “emotional” (Gazzillo, 2023). Given that they store the contingencies that our mind has detected on the basis of our experiences, beliefs can be formulated according to an “if…then” format.

The beliefs that we develop during our childhood are shaped by the emotional and cognitive peculiarities of our psychic functioning during the developmental period. This includes childhood egocentricity and a proclivity to attribute unrealistic responsibilities to the self. Beliefs during this period of development are also influenced by children's dependency on their caregivers and their tendency to think that caregivers are always right and know the truth, so that in cases of disagreements, children think that they are morally wrong or inadequate and their caregivers are right.

When confronted with adverse experiences and traumas, i.e. with situations where our sense of safety is jeopardized acutely (shock trauma), chronically or systematically (stress trauma) (Fimiani, Gazzillo et al., 2020), we consciously and unconsciously try to understand how we could have contributed to these situations and how we can prevent them in the future. Thus, given the egocentricity and dependency of the developmental period, it is not hard for children to answer these questions by *associating the pursuit of healthy and adaptive goals with a danger for the self or for an important other*, and in doing so, to develop *pathogenic beliefs* (see also Dimaggio et al., 2020). Beliefs such as these are pathogenic because they support the inhibition of our attempts to pursue healthy goals and/or link such pursuits with feelings of fear, shame, and guilt. According to CMT, these beliefs shape both our personality and psychopathology.

## 3 CLINICAL AND EMPIRICAL DATA ON GUILT ACCORDING TO CMT

CMT has conceptualized five broad “classes” of pathogenic beliefs that form the basis of irrational feelings of guilt and shame: *self‐hate* (Faccini et al., 2020; Kealy and Gazzillo, 2024; Leonardi et al., 2023), *survivor guilt* (Faccini et al., 2020), *omnipotent responsibility guilt, burdening guilt* (Gazzillo and Leonardi, 2023; Leonardi et al., 2023) *and separation/disloyalty guilt*. The case that we will illustrate later will allow us to observe several clinical manifestations of *burdening guilt* and *separation/disloyalty guilt*. Burdening guilt refers to the pathogenic belief whereby expressing one's own emotions, needs and requests and being spontaneous is associated with burdening other people. This type of guilt‐related pathogenic belief is the outcome of developmental experiences in which children felt neglected, felt that their parents were burdened by their way of being, needs and feelings or, similarly, were too overwhelmed by personal or family issues, and thus unwilling or unable to respond to their children's needs. Burdening guilt is associated with depression, shame, anxiety, suicidal ideation, and attachment insecurity (Gazzillo and Leonardi, 2023).

Separation/disloyalty guilt refers to the pathogenic belief that one can hurt loved ones by distancing physically or psychically from them. This belief may stem from a family environment that inhibited or appeared hurt by the independence, physical distance and differentiation of their children in terms of choices, values, interests, and opinions. Separation/disloyalty guilt is also associated with altruistic behaviors (Faccini et al., 2020).

The multidimensional conceptualization of interpersonal guilt offered by CMT, and validated empirically, expands our understanding of how problematic guilt and shame arise developmentally and manifest clinically. Moreover, this conceptualization emphasizes the role of pathogenic beliefs––reflecting early attempts to adapt to adversity––in determining and maintaining such phenomena. Thus, when encountering patients who struggle with shame‐related and guilt‐related problems, it is important to consider which kind of guilt is most prominent and the nature of pathogenic beliefs involved. Similarly, this perspective suggests that presentations of generalized shame may represent self‐hate pathogenic beliefs that perpetuate a chronic sense of badness and undeservingness, or may be a form of self‐punishment deriving from other kinds of guilt. For example, a person whose parents were burdened by chronic shame can develop chronic shame because s/he would feel too guilty to be happier than her/his parents (Weiss, 1995). Understanding the particular manifestation and expression of these different forms of guilt and shame for a given patient can have significant implications for the therapy process.

## 4 PSYCHOTHERAPY PROCESS ACCORDING TO CMT

According to CMT, patients come to therapy because they want help in achieving the healthy and adaptive goals that are obstructed by the pathogenic beliefs that they developed to adapt to their traumas and adverse experiences. To do so, they need to become conscious of and disprove these pathogenic beliefs and develop better mastery of their traumas.

To disprove their pathogenic beliefs, patients test them within their close relationships, and in particular in the relationship with their therapists. *Tests* are communications, attitudes, and behaviors––instigated largely unconsciously––aimed at disproving pathogenic beliefs. They can also be seen as an (unconscious) attempt to understand how safe is to pursue their healthy and adaptive goals within the therapeutic relationship. Tests may also serve as a way of better mastering their traumas by recreating scenarios within the therapy that are similar to their traumatic situations, with the unconscious hope of achieving a different outcome through the therapist's responses (Gazzillo et al., 2019). It is possible to differentiate two different testing strategies: transference testing and passive‐into‐active testing. We will focus on transference testing since this was the predominant strategy used in our case illustration, presented below. Transference testing is characterized by the patient's vantage point as a traumatized self while attributing to their therapist the role of their (potentially traumatizing) parent. Transference tests can be mediated by communications, attitudes or behaviors that indicate either the patient's compliance or noncompliance with the pathogenic belief being tested.

The following example illustrates these testing strategies. Livia developed the belief that she was a burden to other people because her parents had difficulties in dealing with her strong emotions. To test this belief, Livia could engage in one of the following approaches.

(1) Avoid showing strong emotions in therapy, or describing herself as doing so in other relationships, even if these emotions would be justified by real events in her life, with the hope that the therapist could help her understand that she has the right to show strong emotions and that they are not destructive (i.e., burdening) for other people (transference test by compliance).
(2) Show (or describe herself as having shown) strong emotions, even being rather dramatic in doing so, with the hope that the therapist would not be overwhelmed and would support this emotional expression (transference test by noncompliance).

It is important to underline how patients may test their therapists and important others by adopting particular *attitudes*, and in these cases the optimal response of the therapist should be mainly mediated by their attitude. Indeed, in some treatments of patients with severe narcissistic frailties, the attitude of the patient is their main way of testing, and the attitude of the therapist is the main tool that the therapist can use to pass patients' tests because other kinds of interventions, such as interpretations, comments, exercises or homework, are experienced as endangering (Shilkret, 2006). To be therapeutically useful, the attitude of the therapist should be adapted to the pathogenic belief tested by the patient and to the testing strategy adopted by them. And it should be different from the attitude of the traumatizing parents (or other traumatizing figures) and different from the attitude adopted by the patients for adapting to their adverse experiences.

Although from a certain perspective it is possible to hypothesize that patients test all the time, because they are always interested in understanding if their therapists support or contradict their pathogenic beliefs, it is possible to identify several indicators that make more probable that the patient is testing: (1) the patient makes demands or requests of the therapist; (2) the therapist feels pushed to do something; (3) the therapist feels unusually strong emotions; (4) the patient acts in a way that is more absurd, illogical, or provocative than usual.

Several research projects (see, e.g., Gazzillo et al., 2024) have shown that when patients test their therapists, they feel more anxious, but if the therapist passes the patient's tests, patients feel less anxious and depressed, become more insightful and elaborative, can recover new memories and work harder to pursue their goals and feel more involved in the therapeutic relationship. On the contrary, if the therapist fails to pass their test, patients may coach them to help them understand what they are looking for and what they need, or they may change topic, remain silent and their therapy may come to a stalemate.

According to CMT, patients' coaching communications (Bugas et al., 2023), reflect conscious or unconscious communications aimed at helping therapists understand what they need in that moment of the therapy. Coaching can be proactive, indicating what the patient wants the therapist to understand, or it may be reactive to the therapist's behaviors, indicating whether the therapist is in line or not with what the patient needs. Patients coach their therapists throughout therapy, but they may be more likely to do so (1) at the beginning of the treatment, when they need their therapist to understand what they are looking for; (2) before, during or after an important test; and (3) when they need their therapist to change their attitude or approach.

To sum up, according to CMT patients come to therapy with a relatively articulated and mainly unconscious plan to pursue their healthy and adaptive goals, overcome the pathogenic beliefs that obstruct their pursuit of these goals, and better master the traumas that gave rise those pathogenic beliefs. This plan includes having their tests passed by their therapists––supporting their mastery of pathogenic beliefs and traumas––and understanding themselves and their problems from a more positive perspective. A specific procedure known as the Plan Formulation Method (Gazzillo et al., 2022) has been developed and empirically validated to formulate the patient's plan after the first 2 to 10 sessions, and several research projects have shown that therapists' interventions that support the patient in carrying out their plan are associated with patients' immediate and long‐term improvement in therapy (Silberschatz, 2017). Being “pro‐plan” is thus a measure of therapist responsiveness. In this way, from the perspective of CMT, all psychotherapies must be case specific since they ought to be tailored according to the specific plan of the patient (Gazzillo et al., 2022). Moreover, the core of any therapy is to provide patients with the corrective experiences they need and the tools (e.g., insights, information, attitudes) necessary to disprove their pathogenic beliefs––including those underlying problematic guilt and shame––and to better understand themselves and master their traumas.

## 5 CASE ILLUSTRATION: THE CASE OF LIVIA^2^

### 5.1 Presenting problems and client description

Livia was a 26‐year‐old female patient who decided to enter psychotherapy because she wanted to “re‐start her university career and complete it without suffering”, to become less perfectionistic and controlling, to understand if it was possible to have a better relationship with the boyfriend or if that story was doomed to failure, and to avoid other depressive crises. Livia, in fact, had had a severe depressive crisis during her late adolescence and subsequently other less severe depressive episodes.

During the first two sessions, Livia provided her therapist with plenty of information about her *traumas* and the *pathogenic beliefs* developed to adapt to them. She described her father as sweet and caring, but quite anxious and controlling; “he is a scholar and has always given much importance to school; he has always been the primary caregiver both for me and for my sister, and with the passing of time I have realized that taking care of us has been probably more important for him than for us”. She added also that, when she was ten, she gave her parents a lot of stress because of a suspected brain cancer, and it was only after several months that doctors confirmed that her condition was not a pathological one. Her mother, on the contrary, was quite “domineering”: much younger than the father, “she has spent several years to take revenge of daddy”. Their relationship started when her parents were both quite young and, given their age difference, the father treated the mother as a “little girl” and had affairs with other women. When Livia was a child, her mother was often absent because of her job. Her mother used to love her daughters with a “tough love”: she wanted her daughters to behave well”, and on one occasion the mother did not talk with Livia for “five long days” in response to a minor mischief.

Livia had not enjoyed studying starting from primary school and had always felt it to be a difficult obligation: her mother was not sensitive to her complains about the necessity to study, stressing that “this is what you must do in any case”. By contrast, her father's solution to “the study problem” was to sit down with her and help her study. When she was 11, her father asked her: “Why don't you engage yourself to the fullest at studying?”, and Livia replied “Because if I fail your disappointment would be too strong”.

It was on the basis of these relationships that Livia started to develop the belief that she had to be a “good student and a good girl” to please her parents (disloyalty guilt), and that her needs and wishes could easily be a burden for other people (burdening guilt).

When Livia was 15 years old, a major trauma occurred in her life: she was raped by a former boyfriend whom she had left a few weeks earlier to start a romantic relationship with another boy. They had been together for few months, and their relationship was “push and pull”; from her perspective, his violence was a revenge for her having never surrendered herself to the boyfriend's quest for her total love. This trauma was a powerful confirmation of her belief that she could not disappoint the expectation of people she was close to (disloyalty guilt).

During the first week after this trauma, Livia was supported “in any way” by her sister in mastering what had happened and in dealing with her everyday life, but for reasons she could not understand, her sister then decided abruptly to withdraw her support, saying that she did not “want to know anything more about you and your life”. The reaction of her sister was felt by Livia as confirmation of the pathogenic belief that her emotional needs were “too much” for other people (burdening guilt).

The violence she suffered and the subsequent abandonment by her sister was a “watershed” in her life: she started “to freak out and have great problems at school”, with fights with her mother becoming “the norm in our house”. Livia remembers one time when her mother said to her with a mocking and annoyed tone that “she was always nervous”, and another time when the mother said to the father: “It would have been better if we had left her at the hospital when she was born”. Statements such as these only exacerbated and reinforced her burdening guilt.

After a major fight with her parents, Livia started her first psychotherapy, that she left after few sessions because her therapist was not able to deal with her silence. A similar outcome ensued with “two or three other attempts” with different therapists.

Years later, Livia decided to abandon her studies in university because her difficulty managing the “pressure of having to study for exams I was not always interested in” caused her a “depressive episode”. She felt that she had to study perfectly for every exam, that she had to study every day, and that she had to pass every exam at the first attempt and with the maximum grade. This was all very painful, and she had felt constricted in living a life that she did not want but could not abandon. To Livia that life did not make any sense for her, but she could not disappoint all the people of her life. Livia was hospitalized at that time and received the diagnosis of bipolar disorder along with pharmacological treatment that “transformed her into a vegetable” such that one of her relatives, a medical doctor, questioned the diagnosis and pushed her not to take the medicine anymore. She followed that advice and subsequently she slowly recovered. At that point, her family started a family therapy that she “felt could have saved her life”. However, the therapist decided that “the work was done” when Livia was able to talk about the rape, which left Livia feeling deeply disappointed as she had thought that “that was just the beginning for me”. She decided to look for a job and start working (the same job she was doing when she started her CMT therapy).

Finally, Livia had been involved since her childhood in a semiprofessional sport activity she was very successful at, and her trainer was a woman who was very “severe and demanding”. The trainer had lost the opportunity to become a professional athlete because of a severe accident she had been victim of, and may have regarded Livia as a figure through whom she could potentially obtain the successes that she herself had been forced to renounce. However, after several years Livia also abandoned this activity: she could no longer tolerate the pressure to be perfect and to succeed in order avoid disappointing her trainer. Thus, even the relationship with her trainer had ended up supporting her disloyalty guilt belief.

### 5.2 The therapist

Livia started a psychotherapy with the first author of this paper, a Control‐Mastery therapist with a psychoanalytic background and 20 years of post‐training clinical experience. The therapist's professional experience over the previous 20 years had encompassed relational difficulties and problems in self‐esteem regulation, the very kinds of problems that Livia had presented with.

### 5.3 Case formulation

After the first three sessions, the therapist was able to formulate Livia's plan: her main *goals* were to restart and complete her academic career, that she had interrupted a few years before; to be less perfectionistic and controlling; to feel deserving of receiving more emotional and practical support from her boyfriend; and to be able to argue constructively with her boyfriend. Moreover, she wanted to improve her self‐esteem and to avoid further depressive crises.

According to the therapist's formulation of her plan, Livia's problems derived mainly from two *pathogenic beliefs*: she believed that, if she had communicated her emotions and needs to other people, these people would have felt burdened; and she believed that if she had been spontaneous and had tried to satisfy her needs, she would have hurt and disappointed other people. In other words, she suffered from burdening guilt and disloyalty guilt.

After her first three sessions, Livia and her therapist decided to begin working in a CMT psychotherapy at a frequency of two sessions per week.

### 5.4 Course of treatment

The very first thing that Livia said to her therapist was that she was looking forward to starting to work with him because, several years before, she had seen one of his lessons at university and she had immediately realized that “you were the person with whom I wanted to work in therapy; just as it happens when you find the man of your life”. Livia remembered in particular that she was “struck” by one thing that the therapist said to his students: “If you do not attend the lessons, it will be difficult for you to pass the exam”. In this way, Livia clarified her idea of her therapist as a person of value but very demanding: someone you must be careful not to disappoint.

The therapist, on his side, felt quite at ease with Livia. After having formulated her plan and shared with her which, in his opinion, were her goals, pathogenic beliefs and traumas, the therapist thought that it would be very important to help Livia feel that her feelings and needs were not a burden and that she was free to do what she wanted without being afraid of disappointing him. In other words, the therapist interpreted Livia's presentation both as a transference test by noncompliance of her burdening guilt (“you are something like the man of my life”) and as a transference test by compliance of her disloyalty guilt (“I am afraid that if I do not do what you say, I will disappoint you”). His response was saying, with a smile on his face, “Let's see what we can do”. The therapist thought that the best attitude he could adopt with her was a warm, light and “laissez‐fare” one.

From a diagnostic perspective, a couple of times in the first month of treatment Livia asked her therapist if she met the criteria for a specific diagnosis of mental disease, which the therapist thought was a transference test by compliance of her burdening guilt: “Am I so disordered to deserve a psychiatric diagnosis?” Both times he replied: “No, you only need some help to become less demanding with yourself and to feel more at ease in showing what you need”. He was sincere because, notwithstanding her frequent fights with the boyfriend, her perfectionism, and her difficulties in completing the activities she started, she was able to love, work and have close friendships. Moreover, she did not present with psychiatric symptoms and did not meet the criteria of any clinical or personality disorder.

Livia's therapy lasted for two and a half years and can be divided into two broad phases: in the first phase, Livia worked mainly to disprove her pathogenic beliefs reflecting burdening guilt, and in the second phase she worked hard, in specific ways, to disprove the pathogenic belief supporting her disloyalty guilt.

Since the very beginning, after having said to her therapist that the main reason for wanting to start a treatment was to find a better balance in her relationship with her boyfriend, and after having told her history and having talked for a couple of sessions about the violence she had suffered, Livia started to talk with her therapist about her relational problems. It was not difficult to find a recurring pattern in their fights: she asked something of her boyfriend or felt hurt by something he had done or said, and then she attacked him, verbally or physically. The boyfriend reacted to that attack, and Livia fought back in an escalation or retreated in a complete silence that could last for days. During the first 6 months of the treatment this pattern was repeated again and again, and many times Livia reached the conclusion that she was “crazy” and felt a deep sense of shame and guilt for how she had behaved. A climax was reached 1 day when, feeling that her boyfriend was not paying attention to her because he was playing with his cellphone, she threw his phone on the ground and broke it before biting him on the shoulder.

The therapist thought that those behaviors were transference tests by noncompliance of Livia's pathogenic belief that her emotions and needs were a burden to other people: she tested this belief both with her boyfriend and her therapist. For this reason, the therapist's strategy was to help Livia not feel so ashamed and guilty by reassuring her and helping her understand that those outbursts were the ultimate outcome of many occasions where she had felt that she needed something that she was not able to ask for clearly, or that her boyfriend was not able to understand. On one occasion, for example, Livia got very angry with her boyfriend because he was complaining about the paucity of the food that Livia had prepared, while Livia that same morning had thought that she would have been happy if *he* could have cooked something, given that she had been working quite hard. But she had not felt like asking her boyfriend to do so, reasoning that he too was tired.

After the incident where she broke her boyfriend's phone, there were no more fights between Livia and her boyfriend until the end of Livia's therapy, more than 1 year and half later: they had learned how to disagree and how to reach a compromise, which had been one of her therapeutic goals. Quoting what she said at the end of her therapy: “Thanks to you (the therapist), I was able to understand that if I had been able to verbalize what I needed anytime I felt to need something, with everyone, without feeling that I was a burden, I would not have had those rage outbursts anymore. You were able to make me understand that my requests were legitimate, that I was legitimated to ask for a suggestion, to ask for help and to ask to my boyfriend to help me with our housework even if he was working more than me. You helped me realize that those requests could feel “normal” and “obvious”, while in my mind they were so difficult to communicate and burdensome.”

With the passing of the first year of therapy, both her relationship with her boyfriend and her relationship with her therapist became for Livia a “secure base”: she resigned from her job and restarted her university attendance. Her goals were to graduate and start a new job connected to her studies. She also wanted to go back and live in a house closer to the center of the city where she had lived before her relationship with her boyfriend. Several years earlier, at the beginning of their relationship, in compliance with one of her boyfriend's requests, she had agreed to live together in his home, in a smaller town outside Rome. But now she felt unhappy with that arrangement and wanted to come back, even if her boyfriend wanted to remain in his little town.

The second phase of Livia's treatment was centered around the problems she had to deal with in attending university. As had been the case in the past, when confronted with the necessity to study for exams, she started to feel “constricted”, to have difficulties in studying, and to feel very anxious when she started doing so. Livia felt that she had to study “everything and perfectly” despite not feeling very interested in most of the topics. When the therapist helped Livia notice that her problem with studying was connected to this sensation of being “constricted and having to do something she did not want to do”, Livia connected these sensations to her violence, while also adding that her problems with studying had been present since childhood. At that point, together with her therapist, in a series of sessions Livia realized how she had felt similarly “constricted” by her trainer and by the “tough” attitude of her mother toward her difficulties in studying (“This is what you must do, there is no escape”). Moreover, she also realized how she had “inherited” the anxious and controlling attitude of her father: everything should be planned in advance and executed with precision. She had to comply with this attitude to reward her parents for all they had done for her, and to avoid disappointing “the expectation of other people”. Thus, Livia developed insight into a fundamental dilemma: if she did not study all day, she felt guilty; if she studied all day, she felt oppressed.

One session in the second year of psychotherapy, a few weeks before Christmas, was very important. Quoting her words: “last time I was totally in crisis, I had cried all day, I had not washed myself before coming here and I was wearing my pajamas under the shirt. I was desperate because I had spent hours on my books without learning anything, and I was thinking that I was spending my days doing something I did not like. I hated this and I was regretting my idea of going back to university. And I had the impression that you (the therapist) were not really understanding me, and that you were mistaken in your attempt to “minimize” what was happening by showing me that there was nobody who was constricting me, and to make me smile by stressing that there was no catastrophe in front of us. During the session and soon after it I did not feel understood by you as I usually do, and when I was in my car going home, I thought that in the following session I would have said *this* to you. But, strange enough, during the following days I slowly realized that that was the only reaction I really needed, not someone who complied with me and helped me swim in my desperation, but someone who helped me realize that my reaction was too strong, and that I was not reacting to the problem of studying per se, but to all the experiences and meanings that were behind it. And I realized that I experience the pressure of these expectations and of my perfectionism in all my life, every day, in my friendships, in my love life, in my family. I always have to be on time, and I always have to reply to text messages. And it is always “all or nothing”: I totally comply with the other person, or I get angry for every little thing; I am completely understanding with the other person, or I disappear. Now I think that the problem is that I always want to be the better version of myself, and this cannot work because it forces me to be in a way that a lot of times does not correspond to what I feel, so that I had to reject everything. It is something similar to what happened with my boyfriend. And now I feel not in crisis anymore”.

During that “crisis” session, the aim of the therapist was to help Livia regulate her emotions, mainly by using his attitude more than the content of his communications. Both the patient and the therapist were afraid that Livia was going to have another breakdown that might eventuate in a severe depression. After what Livia said in the following session, the therapist thought that Livia was testing both her burdening guilt and her disloyalty guilt in the transference by a strategy of noncompliance: her anxiety and depression seemed to be almost out of control, and she was showing her worst side. And Livia had experienced his warm, relaxed and accepting attitude as useful for disproving those beliefs.

The therapist then decided to give a little advice to Livia: suggesting to reduce the number of hours spent studying, even if she was afraid that she would not pass her exams, and even right before the exams. He suggested that she dedicate some time every day to rest or go out with friends. “Do less” was the motto. Informed by his formulation of Livia's plan, the therapist reasoned that this intervention could help to change her way of experiencing her studying while also offering the possibility to realize that she was able to pass her exams with very good grades without being perfectionistic. Livia agreed, and thus experienced a corrective emotional experience through the therapist's actions.

After that session, important changes occurred: a few weeks later, Livia wanted to reduce the number of sessions from two to one session per week, and the therapist agreed. After a couple of months, she then started to miss one or two sessions every month, each time explaining by text‐message why she was not coming. The therapist thought that her reducing the number of, and missing sessions were transference tests by noncompliance of her disloyalty guilt. Therefore, he decided neither to problematize nor to investigate this behavior. The therapist suspected his hypothesis was correct because every time Livia came to her sessions, she said that was feeling fine, that her relationship with the boyfriend was going smoothly and that she was being able to study without problems and pass her exams with very high grades. This was Livia's way of coaching the therapist so that he could understand that they were working well.

In the following period, the sessions became more similar to a chat with a friend than to a “psychotherapy session”, and their atmosphere was light and warm. Livia was working on her disloyalty and burdening guilt by being a very imperfect but warm patient. Canceling at least half of her sessions was her unconscious way to prove to herself that she could be herself and do what she wanted without disappointing her important others. And to pass these tests, the therapist had simply to accept this behavior with a serene soul.

One year after the “crisis” session, another small crisis happened, and for the same reason. Another exam series was forthcoming, and she was yet again experiencing, though less dramatically, the same sense of constriction. However, at the end of that session, while leaving the therapist's room, Livia said to him: “I have understood my real problem. The real problem is that I feel I must complete university because I do not want to disappoint daddy. He has spent all his life studying, he has always loved studying much more than working, and he has always been the person who has really taken care of me. But I hate studying. What I want to do is to become a mother and be a housewife!”. The therapist was surprised and realized that he himself felt a bit disappointed by the idea that Livia, who was talented and clever, could once again abandon her academic career. But it was clear to him that she was proposing another transference test by noncompliance of her pathogenic belief that if she would be herself, her important others would have been disappointed. For this reason, he decided to reply that: “It is very important that, eventually, you have been able to really understand what you want for your life. This is the single most important thing that a person must understand. So, do what you think is right for yourself”.

The following week Livia went to her session and was in a much more relaxed mood; she and her therapist laughed a bit about her idea that her life project was to become a “mother and a housewife and nothing more”. Livia said that she had decided to graduate, in any case, and to do a master's degree after graduation.

A few weeks later, Livia communicated to her therapist that she had been pregnant for a couple of months but was waiting to be sure that everything was going fine before sharing this news with him. “I can be both a good mother and housewife and an accomplished professional”, she said smiling.

At that point, Livia and her boyfriend decided that it would have been better both for themselves and for their child to go back and live in Rome, as Livia wanted, and Livia decided to stop her therapy, “at least for the moment”, because she needed to spend her money for their new house. She felt happy and knew that she could keep in touch with the therapist and to visit him in the future if she needed to. The therapist, interpreting this as another transference test by noncompliance of her disloyalty guilt, replied that she could do this anytime she wanted.

### 5.5 Outcome and prognosis

Three months after the termination, Livia wrote to her therapist that “I am as serene as I have never been in my life. I have been able to use in different domains of my life our motto “to do a little bit less is ok” and this makes me feel much more at ease. Sometimes I have to remind this to me, but this is what I need. Our weekly 50 min were “my space” and after them I felt mentally lighter as I have never felt before. I could say and do anything I wanted without feeling judged. I could be myself without feeling judged, ashamed, or embarrassed. And I have always known that you were sincere when you said to me that I could have called you or sent you a text message anytime I would have wanted. I have always felt, since the beginning of our work, that you would not have rolled your eyes or snorted if you had seen my name on the phone. My life is going fine, the pregnancy is going well, and we are remodeling our new house so that it will be ready when my child will be born. I will come and visit you soon”.

### 5.6 Clinical practices and summary

The case of Livia shows several points that are central in how CMT conceptualizes and treats guilt‐ and shame‐related problems. First of all, it was the patient who unconsciously decided what to put at the center of her therapy in its different phases by choosing to start with her history and her trauma, and then dealing first with her problems with the boyfriend; and only after several months did she shift the focus to her problems with studying. This does not mean that in the first year of therapy she had not talked about her problem with studying, but rather that this was not the focus of most of her sessions. More importantly, even when she was talking this during the first phase of therapy, she was doing so mainly to disprove her burdening guilt pathogenic beliefs. According to CMT, it is the patient who unconsciously sets the agenda of the therapy on the basis of their unconscious plan; the task of the therapist is to understand what the patient is looking for and to help them find it.

The second point is the relevance of the therapist's attitude in disconfirming patients' pathogenic beliefs. In the case of Livia, it was not difficult for the therapist to adopt a welcoming attitude with the patient because he genuinely liked her (disconfirming pathogenic beliefs related to burdening guilt). Much more difficult was to respond in a pro‐plan way to the many sessions missed by the patient; to adopt the right attitude toward this issue (disconfirming pathogenic beliefs related to disloyalty guilt) it was very important for the therapist to rely on the formulation of Livia's plan and to be sensitive to the coaching that she gave to him. Similarly, it was difficult for the therapist to pass the test that Livia proposed to him by saying that she had understood that she wanted to be a mother and a housewife and nothing more.

This introduces a third element that is central in a therapy guided by CMT: the fact that patients' tests must be passed, rather than interpreted. Often tests must be passed many times before being interpreted. According to CMT, the core of psychotherapy––and a core mechanism in any successful therapy––is providing patients with the corrective experiences they seek through their tests, trusting in their motivation to have their pathogenic beliefs disproved and to feel safer, and in patients' motivation and ability to develop insights when therapists help them feel safer by passing their tests and adopting pro‐plan attitudes. It was only at the end of the therapy that Livia's therapist said to her that, in his opinion, she needed to see that she could miss so many sessions to really feel free to be herself without worrying about disappointing the people she loved. This does not mean that interpretations played no role in Livia's treatment. Indeed, the therapist's interpretations helped her develop a better understanding of the origins and meanings of her pathogenic beliefs. What Livia found more important, however, was her therapist's relaxed attitude toward her studying, that he did not show any expectation about how she should have worked in her therapy, and his affective warmth and availability.

To sum up, CMT suggests a few key ingredients are involved in helping patients overcome their problems with irrational guilt and shame: (1) the quality of the therapeutic relationship; (2) the ability of the therapist to pass patients' tests and to adopt the right attitude; and (3) the therapist's accurate understanding of and responsiveness to the patient's plan and the patient's coaching communications. Together these offer the possibility for patients to develop a more benevolent and comprehensive perspective on themselves and their own life.

## ENDNOTES

1 With mastery we mean to regulate emotions and sensations deriving from traumas and to make sense of those events by integrating memories of them within the wider network of personal experiences, thereby recovering a sense of safety.

2 The patient has given written informed consent to have this material published and has read and approved how her case is presented. The material, however, has been disguised to preserve her privacy.
